# Data Integration in the Brazilian Public Health System for Tuberculosis: Use of the Semantic Web to Establish Interoperability

**DOI:** 10.2196/17176

**Published:** 2020-07-06

**Authors:** Felipe Carvalho Pellison, Rui Pedro Charters Lopes Rijo, Vinicius Costa Lima, Nathalia Yukie Crepaldi, Filipe Andrade Bernardi, Rafael Mello Galliez, Afrânio Kritski, Kumar Abhishek, Domingos Alves

**Affiliations:** 1 Bioengineering Postgraduate Program of the São Carlos School of Engineering University of São Paulo São Carlos Brazil; 2 Polytechnic Institute of Leiria Leiria Portugal; 3 Institute for Systems and Computers Engineering at Coimbra Coimbra Portugal; 4 Center for Health Technology and Services Research Porto Portugal; 5 Department of Social Medicine of Ribeirão Preto Medical School University of São Paulo Ribeirão Preto Brazil; 6 Community Health Postgraduate Program University of São Paulo Ribeirão Preto Brazil; 7 Academic Tuberculosis Program Medical School of Federal University of Rio de Janeiro Rio de Janeiro Brazil; 8 Department of Computer Science and Engineering National Institute of Technology Patna India

**Keywords:** health information systems, tuberculosis, ontology, interoperability, electronic health records, semantic web

## Abstract

**Background:**

Interoperability of health information systems is a challenge due to the heterogeneity of existing systems at both the technological and semantic levels of their data. The lack of existing data about interoperability disrupts intra-unit and inter-unit medical operations as well as creates challenges in conducting studies on existing data. The goal is to exchange data while providing the same meaning for data from different sources.

**Objective:**

To find ways to solve this challenge, this research paper proposes an interoperability solution for the tuberculosis treatment and follow-up scenario in Brazil using Semantic Web technology supported by an ontology.

**Methods:**

The entities of the ontology were allocated under the definitions of Basic Formal Ontology. Brazilian tuberculosis applications were tagged with entities from the resulting ontology.

**Results:**

An interoperability layer was developed to retrieve data with the same meaning and in a structured way enabling semantic and functional interoperability.

**Conclusions:**

Health professionals could use the data gathered from several data sources to enhance the effectiveness of their actions and decisions, as shown in a practical use case to integrate tuberculosis data in the State of São Paulo.

## Introduction

### Background

One of the key issues regarding health information systems is their lack of interoperability [1]. Systems do not exchange data, and even when they do so, data do not have the same meaning. This situation can lead to disconnected service operations, rework, and poor comprehension of medical terms, which, in turn, can influence the quality of health services due to the increase in medical errors as well as health care costs [2]. In addition, data availability for scientific studies can be limited [3].

Tuberculosis (TB) is a curable disease, but in 2018, it was among the top 10 causes of death, with 1.5 million deaths and about 10 million new cases worldwide [4]. Brazil follows the directly observed treatment, short-course (DOTS) strategy recommended by the World Health Organization [5]. In Brazil, there are at least 8 main health information systems for TB, namely SISTB, ILTB, Hygia Web, Notification and Monitoring System for Cases of Tuberculosis in the State of São Paulo (TBWEB), Notification of Injury Information System (SINAN), Laboratory Environment Manager (sistema Gerenciador de Ambiente Laboratorial [GAL]), SITE-TB, and electronic Sistema Único de Saúde (e-SUS) AB. In some of these applications, health professionals have to reintroduce the same information and, when the patient's historic information is needed, manual processing is required. In a previous work, we studied the quality of the information among the data of 3 of these systems, and we found poor consistency and reliability [6].

In today's web, most of the available content is suitable for human interpretation and is therefore not easily accessible by other machines and systems. The Semantic Web, defined by Berners-Lee et al [7], can be specified as an extension of the current web, with the purpose of adding logic to the content to express the meaning of information, its properties, and the complex relationships existing between different types of data, so that it is possible to interpret the meaning of given data without worrying about the form of representation [8]. The goal is to create an efficient way to represent data on the World Wide Web to build a global database of connected data [9], through the semantic marking of web pages and existing relational databases using ontologies to provide a common meaning. Thus, this work proposes the use of the Semantic Web and a top-level ontology to support the interoperability of the Brazilian public health systems for tuberculosis, as an alternative to other standards, such as OpenEHR [10], mainly due to its flexibility, ease of implementation, and low level of needed intervention in the architecture of existing health information systems. Our work focuses on the specific scenario of Brazil for TB treatment and follow-up using DOTS.

### Interoperability

The ability of two or more systems to exchange information and use it transparently is defined as interoperability [11]. For this, there are standards, languages, and protocols that must be followed, depending on the type of interoperability that one wishes to achieve.

According to the Healthcare Information and Management Systems Society, there are four levels of health information technology interoperability: foundational, structural, semantic, and organizational [12]. Foundational interoperability allows data exchange from one information technology system to be received by another and does not require the ability for the receiving information technology system to interpret the data. Foundational interoperability approaches range from direct connections to databases to service-oriented architectures using, for example, web services. Structural interoperability is an intermediate level that defines the structure or format of data exchange (ie, the message format standards) where there is uniform movement of health care data from one system to another such that the clinical or operational purpose and meaning of the data are preserved and unaltered. Structural interoperability defines the syntax of the data exchange. It ensures that data exchanges between information technology systems can be interpreted at the data field level. Structural interoperability is based on the concept of enterprise service buses, using standards for message formats. In health care systems, HL7 is the reference as a de facto standard [13]. Digital Imaging and Communications in Medicine (DICOM) [14] is another reference regarding the exchange of data between devices and image information systems. NextGen Connect Integration Engine [15] is also a cross-platform engine allowing the bidirectional sending of messages in many supported standards (such as HL7 V2, HL7 V3, HL7 Fast Healthcare Interoperability Resources, DICOM) between systems and applications. Semantic interoperability provides interoperability at the highest level, which is the ability of two or more systems or elements to exchange information and to use the information that has been exchanged. Semantic interoperability takes advantage of both the structuring of the data exchange and the data codification, including vocabulary, so that the receiving information technology systems can interpret the data. This level of interoperability supports the electronic exchange of patient summary information among caregivers and other authorized parties via potentially disparate electronic health record (EHR) systems and other systems to improve quality, safety, efficiency, and efficacy of health care delivery. OpenEHR [16] is a reference regarding semantic interoperability in health care. It is an open standard specification in health informatics that describes the management and storage, retrieval, and exchange of health data in EHRs. Finally, we can also consider organizational interoperability, which is concerned with how different organizations collaborate to achieve their mutually agreed electronic government goals. Concerned organizations need detailed agreement on collaboration and synchronization of their business processes to deliver integrated government services [17]. Contributing to the best practices of integrating systems and providing the basis for the organizational interoperability, Integrating the Healthcare Enterprise (IHE) profiles offer a common framework to understand and address clinical integration needs. IHE profiles are not just data standards; they describe workflows, which makes them more practical for use by health care information technology professionals and more applicable to their day-to-day activities [18].

Semantic interoperability can also be achieved with the Semantic Web. The basic blocks that define the Semantic Web are a standard data model, query protocol, and set of reference vocabularies. W3C standards and definitions, such as the Resource Description Framework (RDF), SPARQL Protocol, and RDF Query Language, and ontologies refer to these basic blocks, defined as a description language and data model, query protocol to obtain data stored in RDF, and formal representation of a given knowledge, respectively [19]. Ontologies can be defined as a formal representation of knowledge in a specific domain [20], aiming to formulate a rigorous and exhaustive conceptual scheme. In turn, Web Ontology Language is a semantic markup language for publishing and sharing ontologies, designed to describe classes and relationships between them [21].

In the Brazilian scenario, Ministry of Health Ordinance 2073/2011 regulates the use of interoperability standards in the scope of the Unified Health System (SUS) and the supplementary health sector [22] to guarantee functional and semantic interoperability for health information systems. Specifically regarding TB, follow-up of TB cases involves the filling of several registry instruments standardized by the Ministry of Health, such as the Individual Notification Form (Formulário de Notificação Individual [FNI]), Record of Directly Observed Treatment, and Record of Treatment and Follow-up of Cases of Tuberculosis, in addition to the electronic medical record, TBWEB, SINAN, SITE-TB, GAL, and e-SUS AB at a nationwide level. Other local systems are also involved, namely Hygia Web, SISTB [23], and hospitals’ information systems, which are, respectively, governmental, state, and regional health information systems.

SISTB stores and centralizes information about the patient, treatments, examinations, and hospitalizations in the municipality of Ribeirão Preto. HygiaWeb is the public health management software of Ribeirão Preto city, which connects many levels of the local health care system. TBWEB is software developed by the government of the State of São Paulo for epidemiological surveillance. SINAN is software used nationwide to notify of every new case of certain compulsory notification diseases that are stipulated by the national government. GAL allows the management of routines and the monitoring of the steps for conducting exams, containing data that could be associated with the records of exams stored in the SISTB. SITE-TB is a platform that supports the notification of all prescriptions for treatment that does not involve the drugs that are generally used for drug-resistant TB (ie, rifampicin, isoniazid, pyrazinamide, and ethambutol). E-SUS AB is the basic attention health management software at a national scope.

These numerous systems with different technologies, data formats, and semantics generate difficulties in the follow-up of the patient since they can create duplicity, losses, and contradictory information [6].

In the next section, we present some key works in the area of interoperability using the Semantic Web.

### Related Work

At first, Lopes and Oliveira [24] used the metaphor of closed and distributed silos where the health data are fragmented distributed, thus highlighting the rusticity of the software that deals with them. The authors suggested a migration to the Semantic Web model through a framework idealized by the same authors with several resources that allow not only the extraction of data but also knowledge. Valle et al [25], in a similar initiative, advocated for the adoption of the Semantic Web paradigm with technologies widely used in the market, such as Extensible Markup Language and web services, to achieve interoperability in health. The authors defended this approach as it promotes the combination of syntactic and semantic interoperability between applications.

Abhishek and Singh [26] proposed an ontology following the principles of the Basic Formal Ontology (BFO) for the Indian scenario of TB. Hitzler and Janowicz [27] emphasized the increasing use of the Semantic Web. Such growth is possible thanks to the application of the Semantic Web paradigm not being tied to a specific type of knowledge or area. On the contrary, the Semantic Web comes to support environments where interdisciplinarity and heterogeneity are implicit in the routine. In this sense, the increase in the number of conventions and people interested in the subject is justified, whether they come from the academic or commercial milieu.

Ogundele et al [28] developed an ontology for representing, consolidating, and structuring knowledge about the factors that influence treatment adherence behavior in patients with TB. The repository created can be used to find potential factors affecting TB treatment adherence in similar communities that were used in the study, generating its risk indices and also helping the monitoring of patients and their follow-up.

Heterogeneity between conceptualizations must be resolved before handling the term-level syntactic heterogeneity so that semantic interoperability can be conducted in an effective way. In the proof of concept by Gonçalves et al [29], an experiment was conducted that provided evidence that the electrocardiogram ontology can be effectively used to support the design of other interoperable versions that refer to electrocardiograms, such as the HL7 Annotated Electrocardiogram. The authors also affirmed, through their results, that their method can also be applied in other domains.

Kumar [30] discussed some vocabulary as well as how it can be used to achieve interoperability between applications. However, he also discussed the issues surrounding the privacy and interoperability of applications that use the Semantic Web. Also, according to Zenuni et al [31], mapping proprietary formats in ontologies is a complex and intense task, and the maintenance of ontologies is one of the delicate points.

Despite these challenges, some key practical case studies must be referenced. Belleau et al [32] presented the Bio2RDF project using Semantic Web tools to cluster biomedical knowledge extracted from numerous databases. McMurray et al [33] developed a conceptual model of regional clinical electronic exchanges between health care. The ontology allowed visualization of the model and instances by the computational model and was used to validate a subset of the collected data using a different database, although it still had a related database with the interests of the research. Jiang et al [34] described the development of a tool to solidify the definitions recommended by the International Classification of Diseases, version 11. The classification generated by their work through a governance model that involved expert consensus, collaborative and distributed validation, and support allowed these parameters to be optimally tuned. These classifications were then compared with other values found in the Unified Medical Language System and the Systematized Nomenclature of Medicine - Clinical Terms. The compilation of the results was submitted to specialists for evaluation and effective assignment of the degree of usability. Finally, Abhishek and Singh [26] created an ontology to assist the decision-making of managers of the National Indian Tuberculosis Control and Management Program. The basis of this ontology was the BFO, characterized as a meta-ontology that allows the hierarchy and correct division and classification of the entities of the domain that one wishes to represent. This approach facilitates possible mapping between other ontologies that make use of the same meta-ontology for their construction as well as guarantees semantic consistency for application of knowledge extraction algorithms based on Semantic Web Rule Language. Several examples of queries were demonstrated in said work, proving the robustness of the solution adopted.

The next section presents the proposal of an interoperability solution for the Brazilian public health system supporting TB.

## Methods

The cornerstone of a Semantic Web solution is its underlying ontology. Thus, the first step was the development of an ontology considering the clinical TB concepts; the different existent systems FNI, TBWEB, SINAN, SISTB, HygiaWeb, e-SUS AB, and GAL; and the concepts related with TB and DOTS. DOTS is the international strategy for TB control recommended by the World Health Organization that has been recognized as a highly efficient and cost-effective strategy. As already mentioned, this is the strategy adopted by the Brazilian government.

**Figure 1:**
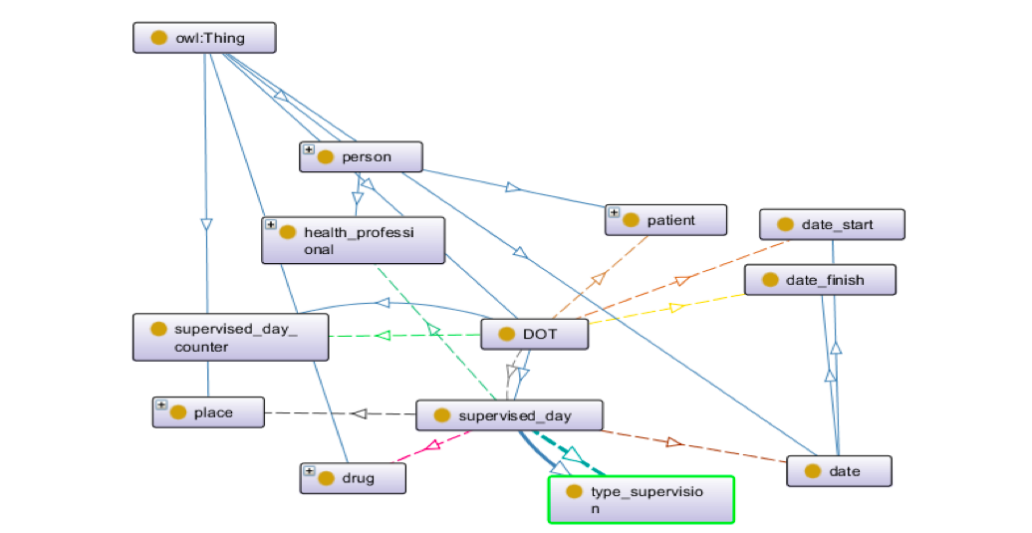
The ontology for directly observed treatment, short-course (DOTS) to support interoperability in the Brazilian public health system.

**Figure 2:**
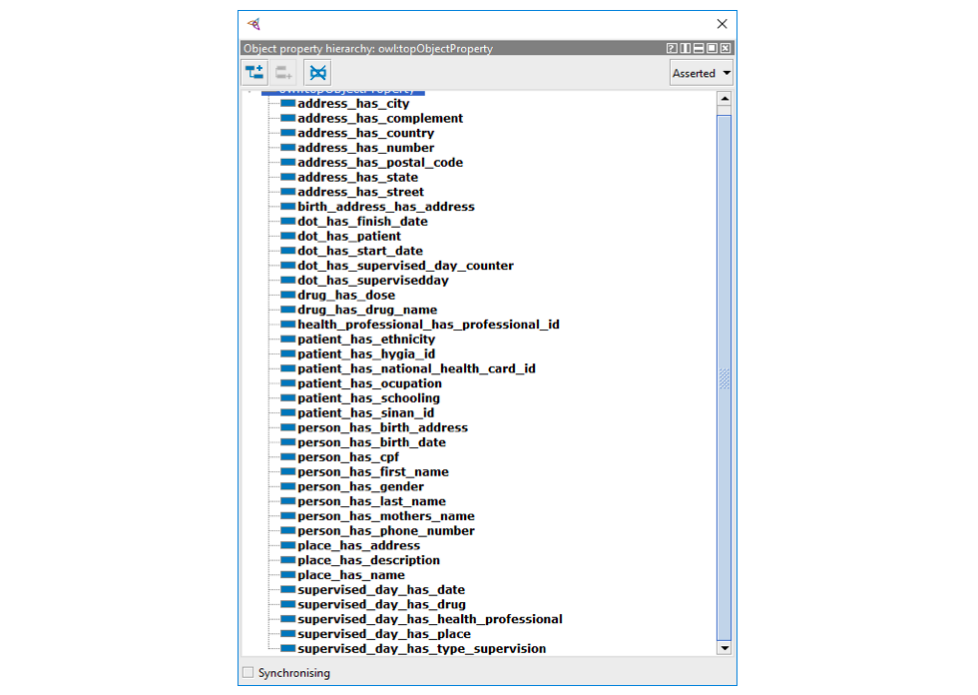
Object properties related to directly observed treatment, short-course (DOTS).

The entities of the ontology were allocated under the definitions of the BFO, which is a top-level ontology initially developed for use in scientific domains such as biomedicine. BFO sees reality regarding a top-level division of all particular entities (individuals) into the two disjoint categories of continuant and occurrent. Continuant entities include objects, attributes, and locations and are contrasted with occurrence entities, which include processes and temporal regions. Processes happen in time and so have temporal parts. Continuants, in contrast, exist in full at any time in which they exist at all. Because it is an upper-level ontology of a fundamentally realistic methodology and has a high level of representation, it allows the mapping of several entities, processes, and their respective functions and characteristics within a temporal space, standing out over other ontologies that only take snapshots of these situations. Given our interest in mapping terms from both medicine and administrative areas, such top-level ontology is an excellent alternative. Figure 1 presents the resultant ontology for DOTS in the Brazilian health policies scope, and Figure 2 represents its object properties.

**Figure 3:**
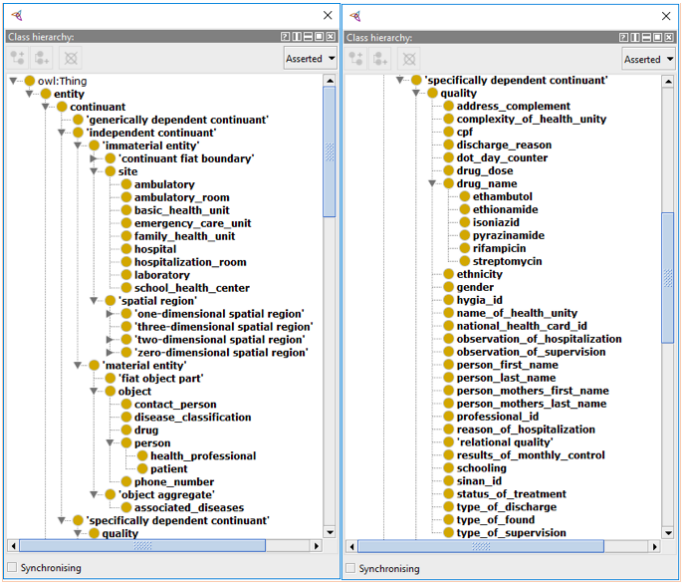
First part of the tuberculosis entities mapped into the Basic Formal Ontology (BFO).

**Figure 4:**
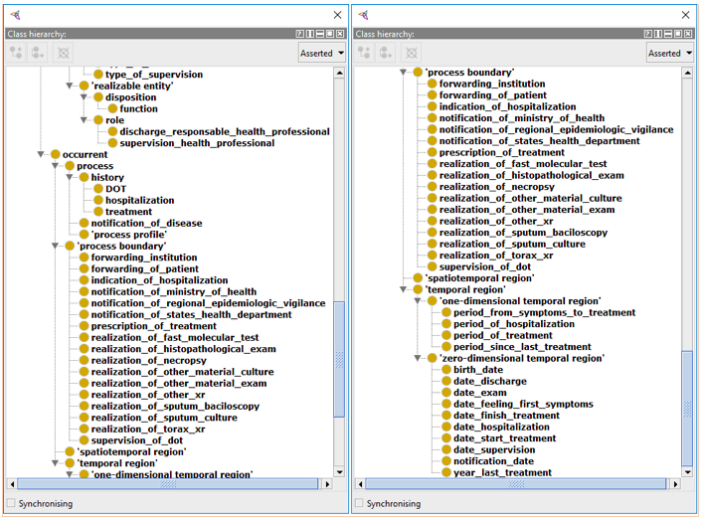
Second part of the tuberculosis entities mapped into the Basic Formal Ontology (BFO).

**Figure 5:**
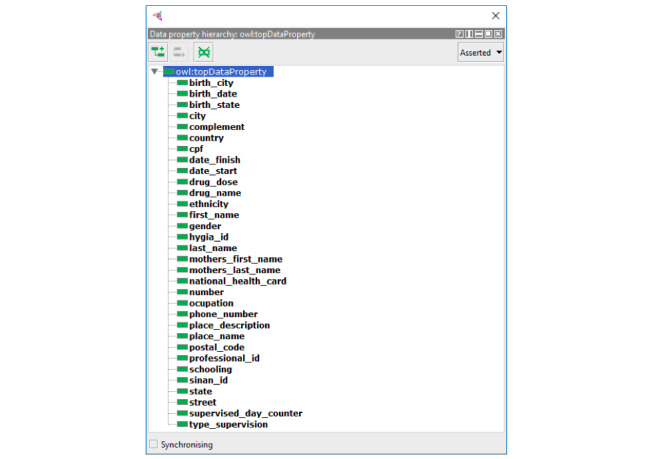
Translated terms that allowed the interchange of data between the applications.

Abhishek and Singh [26] demonstrated the creation of an ontology for the Indian scenario of TB, serving as the basis for the development of the ontology presented in this work. When using the same framework to represent the Brazilian scenario, the mapping ontology-ontology was facilitated. This is because BFO already classifies these terms, making the stage of meaning abstraction trivial and necessitating only the relation of the terms with similar meanings. By eliminating the step of meaning abstraction, an inherent subjectivity burden that can lead to errors in the mapping of one ontology to another, is also removed. Figures 3-5 are excerpts of the concepts that were mapped into BFO.

The mapping of terms in the BFO structure presented considerable difficulty, given the philosophical complexity involved in the definitions and the degree of comprehensiveness we chose to take. It is clear that the granularity of the actions specified in the construction of the ontology can grow as the engineer wants. However, for this work, the resulting formalization shown in Figures 3-5 was the result of consensus among the authors and judged sufficient to support interoperability between the proposed systems.

The key concepts to achieve interoperability between systems and the recovery of DOTS data are described in Figure 5. Patients’ personal data (address, birth date, Natural Persons Register [Cadastro de Pessoas Físicas, CPF], mother’s name) and health-related information (national health card, HYGIA ID, SINAN ID) were described. The transitivity characteristics of the retrieved data, together with the semantic interoperability guarantee provided by the ontology, allowed the merging of the patients' data from the participating applications. This increases the relevance of the information available to the health professional who needs to make strategic and clinical decisions.

This ontology allows the marking of all the necessary data to reach interoperability between the systems previously mentioned.

Data can be obtained through queries on endpoints that support the SPARQL language, as well as any data source semantically marked with the ontology (websites, text documents, spreadsheets, RDF). In the latter case, however, there is a need to process these data to fully exploit the information that does not occur in data returned directly by the SPARQL endpoints. A challenging issue concerns the compatibility of this proposed solution with legacy databases. Such concern is justified by the immeasurable value of knowledge accumulated during several years of care in health services. In this sense, an alternative was sought to address performance and compatibility concerns with relational database management systems. The tool chosen was the D2R Server [35,36], which allows the establishment of a virtual database based on a given ontology and the execution of SPARQL queries on the legacy database, returning the desired information, which are the final values itself, in RDF format and used in a Semantic Web paradigm. This solution, invoked via an application programming interface, contributes in a positive way to reduce the impact caused by the paradigm shift from legacy systems to the Semantic Web. With this approach, there is no need to treat the whole database so that it becomes usable and consumable by web-based semantic applications. Then, this framework allows data exchange between legacy and Semantic Web–enabled applications.

## Results

**Figure 6:**
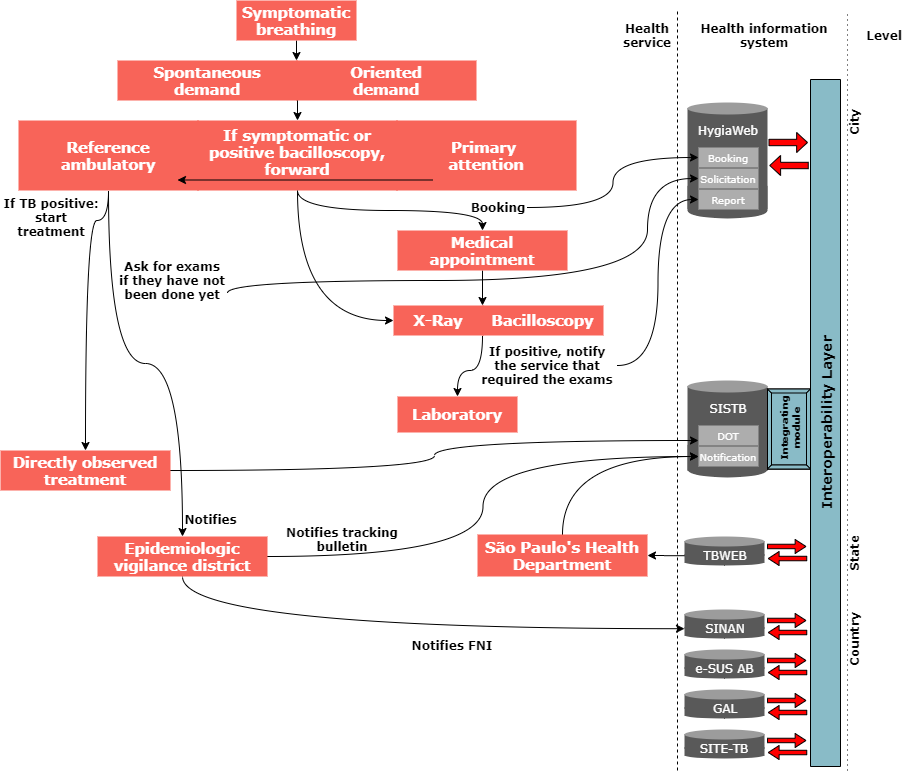
Information flow for tuberculosis treatment after implementation; adapted and improved from [37,38]. DOT: directly observed treatment; e-SUS: electronic Sistema Único de Saúde; FNI: Formulário de Notificação Individual; GAL: sistema Gerenciador de Ambiente Laboratorial; SINAN: Notification of Injury Information System; TBWEB: Notification and Monitoring System for Cases of Tuberculosis in the State of São Paulo.

In Figure 6, the implementation of the interoperability layer between FNI, TBWEB, SINAN, SISTB, HygiaWeb, e-SUS AB, SITE-TB, and GAL, which allows transparent information exchange is presented. The interoperability layer was based on the Semantic Web paradigm and standards preconized by W3C. This paradigm allows the extraction of content from these systems in an optimized way for machines through a web service, opening a range of possibilities for generating useful information for decision making, as specified by Berners-Lee et al [7].

The search for health services occurs when the patient has some symptoms related to the respiratory system. Such demand may be spontaneous or oriented. Spontaneous demand refers to when the patient searches for a general practitioner or pneumologist on his or her own. A demand-oriented search is when the patient is referred to this specialty by the general practitioner, primary care physician, or family physician. The patient is then referred to primary care (if the search for care has not started at this point). If the symptoms corroborate the presumed TB, chest X-ray and sputum examination are requested, and a medical appointment is scheduled. After obtaining the exam results, the patient is referred to an outpatient clinic where the treatment will begin. Treatment is directly observed, and all follow-up data are stored in SISTB. The directly observed treatment is a health policy that intends to closely monitor the evolution of the treatment and patient to increase the effectiveness and success rate of the treatment.

To achieve interoperability, it is necessary to semantically tag participant systems using the ontology previously presented. To do that, HTML pages can be tagged via a Microdata framework [39,40] that extend the HTML specification with specific attributes. Also, a middleware, such as D2R Server, can be configured to expose relational databases as virtual RDF datasets.

Tags refer directly to the terms used in the ontology. This markup format was chosen because it allows the search engines to easily extract knowledge from the fields marked with the tags since the HTML language is a common basis for web applications. The great advantage of this knowledge extraction is that semantic interoperability is already implicitly inserted in the page context page, since the ontology gives every logical structure to which the tagged data is linked, avoiding further work of assigning meaning to the data returned.

In each of those systems, an active SPARQL endpoint service is desirable to allow running SPARQL queries on information stored in legacy databases. Such an endpoint can be provided by middleware, like the D2R Server. This is fundamental for extracting data that have been stored before adopting the Semantic Web paradigm to enable the interoperability layer.

Extracting information directly from marked pages is done through the library Any23, which directly extracts the objects or literals and their tags (corresponding to the ontology). With the extracted data, it is possible to realize several types of SPARQL queries and to incorporate such information in its local database for any queries.

A very simple SPARQL query, where all properties of all patients are returned, is being used as the basis for the incorporation of data extracted from other systems marked with the respective ontology.

It is important to note that this query can be executed on the data of all the marked systems. This guarantees that the returned data have the same meaning, since they were marked with the same ontology and are, therefore, interoperable between the systems.

Such an approach allows data tagged with this same ontology to be fetched on any system (via HTTP requests or SPARQL endpoint). The returned data are then treated and incorporated into the system, if desired, or can be used for ad hoc queries and statistics, allowing rapid decision making by health professionals. In this sense, both semantic interoperability and functional interoperability work in function of data integrality and rapid response sought by health information systems.

**Figure 7:**
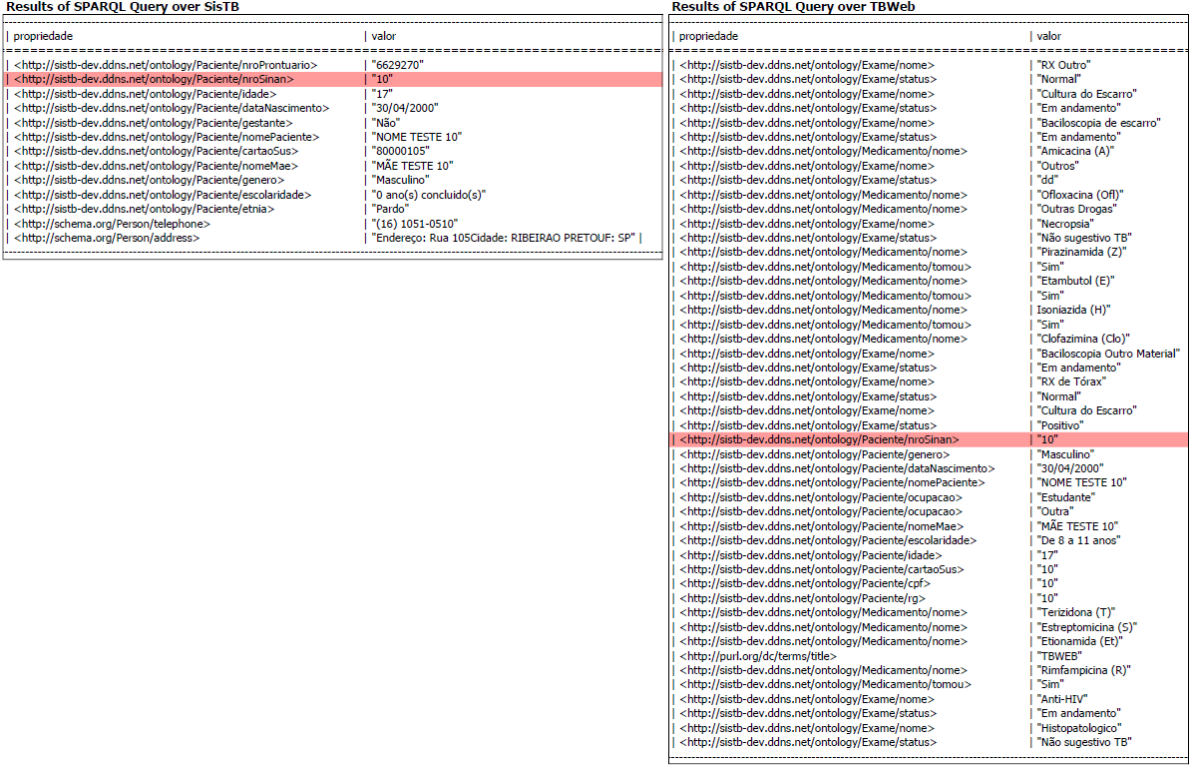
Results of a SPARQL query simultaneously on SISTB and Notification and Monitoring System for Cases of Tuberculosis in the State of São Paulo (TBWEB) for a specific patient with sinan_id=10.

All health information systems in the scope of this work have common data identifying the patient (CPF, National Health Card, birth date). That is, with the semantic marking, it is possible to return the data referring to a particular patient and to aggregate them in a single result or to import only enough data for the decision making, as shown in Figure 7. The intersection of these records represents a snapshot of the current patient’s health situation, corroborating with the integrality in the patient's health care. The retrieval of this information from several health information systems can allow health professionals to make their decisions with more details than would be present if they were using an isolated system. From the managerial point of view, data aggregation for the accomplishment of demographic studies is also improved. The development of more specialized public health policies with better effectiveness is also facilitated since the availability of information on most patients is increased.

A key challenge is to reach interoperability with other standards, such as HL7, OpenEHR, and IHE profiles. In the case of HL7, a viable approach is deploying a middleware capable of translating extracted data through semantic markup into HL7 messages (V2.x or V3.x). Plastiras and O’Sullivan [41] published their work with similar development of a middleware to interoperate data from personal health records to EHRs. However, our proposed middleware receives as input the extracted data of semantically tagged applications, constructs the messages, and forwards them to the recipients. In this scenario, an effort must be made to map the entities of the domain-specific ontologies in the pre-specified fields recommended by the HL7 standard.

**Figure 8:**
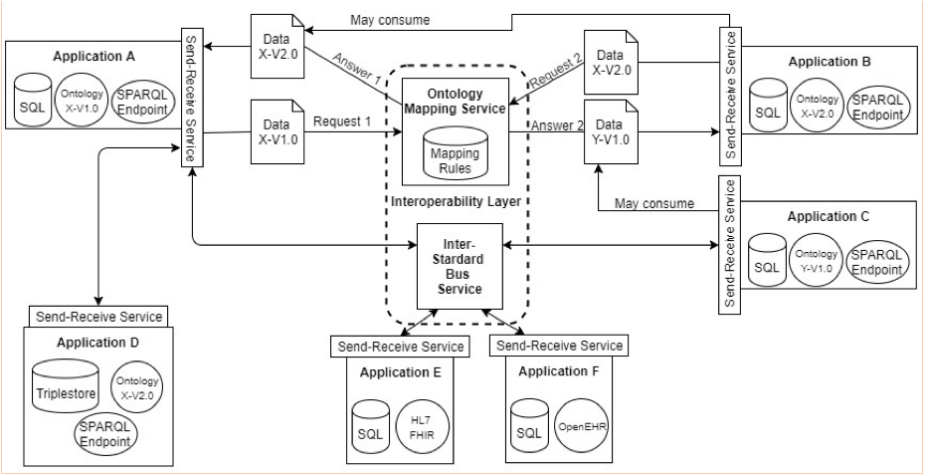
Architecture for interoperability between applications that use the Semantic Web and other standards (ie, HL7 FHIR and OpenEHR).

Such an effort would be similar to the mapping of which entities match a given previously specified OpenEHR archetype. That is, to interoperate systems that use the Semantic Web with systems that use the OpenEHR standard, it is necessary to ensure that the archetypes are fully represented by entities of an ontology. The reciprocal is also true since the mapping of archetypes into entities of an ontology is also extremely necessary. Such processes are exemplified in Figure 8.

As a recent result of the following proposed architecture, it is valid to cite the work of Pellison et al [42]. Their work presented a proof of concept regarding TB data integration in the State of São Paulo, Brazil, using Semantic Web resources, such as SPARQL queries and RDF. Throughout a federated query, data were simultaneously obtained from TBWeb, the state governmental system, and SISTB, the regional TB information system used mainly in the city of Ribeirão Preto (Brazil). By doing this, it was possible to combine data from both sources with aggregated semantic value.

Demographic data were used to push points on a map and to compare values among datasets, which included latitude, longitude, pregnancy situation, age, gender, city of notification, federative unity (state), schooling, and ethnicity. Users were able to obtain information about notification of TB cases using filters available in the interface of the map. The search result was drawn as a heatmap, according to the municipalities that have notified TB cases.

This work, as a proof of concept, calls attention to the importance of working in solutions that could improve the quality of data in the health field and daily activities of health professionals.

## Discussion

### Principal Findings

This study presents an ontology based on the BFO meta-ontology to support TB-related data in Brazil. The construction of the BFO-based ontology benefits from the fact that it is easily related to other ontologies built on the same framework. This allows different ontologies to be related in an easier way because their entities are organized systematically and hierarchically according to their semantic meaning advocated by the same meta-ontology. This interontology relation has great potential because it allows marked data to be shared among the institutions by carrying their respective semantic values and broadening their potential of multicentric research. Currently, there are initiatives to map terms and construct ontologies related to the treatment of TB in other scenarios, as quoted by Abhishek and Singh [26]. As the present work was carried out based on the same meta-ontology and due to the interontology relationship, the possibility of data transposition between the marked systems is feasible and can be interesting for both parties, thus allowing semantic interoperability.

Although solutions like OpenEHR makes domain semantics a central concern, it is optimized to provide a data platform with a stronger focus on the persistence of data, with data exchange a secondary focus. OpenEHR uses a large set of complex archetypes (ie, the model or pattern) for the capture of clinical information, which are designed to provide a maximal set of data elements. This breadth and depth inevitably bring a level of complexity. Semantic Web, on the other hand, delivers more flexibility through the use of ontologies as models to represent health domain data. As OpenEHR archetypes, ontologies can be reused, extended, and adapted to specific demands (*mutatis mutandis*) [43] and can easily be applied to a health information system without so much effort. By using tools designed for the Semantic Web to add meaning to data, such as virtual graph (or tuples) repositories, and developing integrated application programming interfaces to perform data exchange, the level of intervention in health information systems is reduced, resulting in more immediate benefits for functional and semantic interoperability.

During the development of the architecture, some challenges and possibilities of fruitful future research were found. All ontologies can be modified, either because of changes in the domain definition or because corrections in their construction and other adjustments considered necessary for the proper functioning of the ontology. However, such changes directly impact the systems marked with it, and special attention is needed for the data that are marked by the ontology. It is necessary to control ontology versions to admit reconstruction of the ontology cycle of life and to trace the meaning of the extracted data in the version in which it was marked. Therefore, it is necessary to find solutions for the readjustment, most easily and intuitively as possible, for data re-marking and to minimize negative impact on the systems. Some work has already been developed in this area, focusing on the prediction of patterns of ontology changes, for instance, as demonstrated by Javed et al [44].

With version control, we also need to pay attention to how to mark these data, since this task requires considerable effort in systems that use ontologies with many entities and terms. Such automatic markup has a small amount of work performed and documented, perhaps justified by the need for specialized markers in the domain for which the respective ontologies were developed. It is still possible to emphasize some initiatives that have been proposed to help developers to carry out the marking in, at least, a semi-automatic way. Among them, we can mention UCCA-App [45], MnM [46], and SemTag and Seeker [47]. The reduction of the markup effort, be it at first or after the publication of a new version of a given ontology, should be a focus of future work, to provide simplified system maintenance that uses the Semantic Web paradigm.

The flexibility in constructing ontologies and the high level of abstraction they possess makes the mapping process for archetypes relatively trivial when one already has an accepted archetype for the foundation responsible for managing the standard repository. It is still important to emphasize that the Semantic Web approach can be incorporated into several IHE profiles so that integration can occur in several areas, being enough to have an ontology that supports all of them and their respective processes.

Interoperability protocol initiatives that are being carried out in Brazil, especially by the Ministry of Health, have a lot of bureaucracy and technical challenges. This means that the implementation of these standards by themselves includes a lot of effort in recoding the already existing applications, the deployment of endpoints to allow functional interoperability, and having the mapping of the scenario accepted by the institution that takes care of the standard (ie, OpenEHR). This means that the time that is necessary to make many health applications interoperable, both semantically and functionally, is much longer than in the Semantic Web approach. This affirmation is supported by the concept of the Semantic Web itself, which allows a much more flexible governance to develop its ontologies. Semantic Web usage has the advantage of having a much more dynamic domain scope governance than other interoperability standards such as the previously cited HL7 and OpenEHR. Such dynamics favor the evolution of terms and adaptation to new trends, something that is inherently recurrent in health, where techniques, new procedures, and clinical protocols evolve every day.

With this architecture in mind, it is possible to expand the initiative of Conecta SUS, established by the Strategic Plan [48] made by the National Health Terminology Center from the Brazilian government. Conecta SUS is an alternative to improve the scenario shown by Rijo et al [49], where the authors show and assess the lack of interoperability through many health institutions in Brazil.

The results present a viable and practical use of this architecture, opening a new horizon of application at any health care level or specialty.

### Conclusions

In this work, research and implementation of an ontology that supports the Brazilian scenario of TB were conducted. Such an ontology was constructed using the meta-ontology BFO for the classification of terms. This formalization will allow the mapping of Brazilian ontology entities for TB among other ontologies that also have used BFO as a model and have similar entities, facilitating semantic interoperability. Upon the ontology that was built, an architecture was developed to allow functional interoperability between applications that store health data related to TB. Applications were marked via microdata attributes with the terms of the ontology created. This markup enables content extraction from multiple applications from a single SPARQL query on the endpoints installed in each application. It is worth mentioning that solutions have been implemented to run SPARQL queries in relational databases and triplestores, thus allowing the maintenance of legacy databases. The example presented in this article shows how data from a given patient can be returned from all applications that had the same enrollment. The returned data maintained their meanings, and semantic and functional interoperability was achieved. A limitation of our work is the mapping of the entities that concern multidrug-resistant TB, extensively multidrug-resistant TB, and the notification application for these cases (SITE-TB) and comorbidities. Future work will include the map of these workflows and other support applications like the National Regulation System (SISREG) and demographic applications from the Brazilian Institute of Geography and Statistics (IBGE).

Despite the ease of using legacy databases, there is a need to improve the services that would facilitate the implementation of this solution in daily practice. Automatic data marking can be an area of ​​study interest, aiding in the effort required to attribute semantic meaning to the data. Other viable examples in the short term would be the implementation of an interontology and intra-ontology mapping service and also an interstandard message bus and routing service. The first one would allow data to be marked with more than one ontology version and consequently be consumed by different applications that use different ontologies. The second one would allow interoperability between applications that use paradigms and patterns other than the semantic web, such as HL7 FHIR and OpenEHR.

## Abbreviations

BFO: Basic Formal Ontology.
CPF: Cadastro de Pessoas Físicas.
DOTS: directly observed treatment, short-course.
e-SUS: electronic Sistema Único de Saúde.
EHR: electronic health record.
FNI: Formulário de Notificação Individual.
GAL: sistema Gerenciador de Ambiente Laboratorial.
IHE: Integrating the Healthcare Enterprise.
RDF: Resource Description Framework.
SINAN: Notification of Injury Information System.
TB: tuberculosis.
TBWEB: Notification and Monitoring System for Cases of Tuberculosis in the State of São Paulo.
